# Why rate when you could compare? Using the “EloChoice” package to assess pairwise comparisons of perceived physical strength

**DOI:** 10.1371/journal.pone.0190393

**Published:** 2018-01-02

**Authors:** Andrew P. Clark, Kate L. Howard, Andy T. Woods, Ian S. Penton-Voak, Christof Neumann

**Affiliations:** 1 Brunel University London, Uxbridge, United Kingdom; 2 University of Bristol, Bristol, United Kingdom; 3 Xperiment, Lausanne, Switzerland; 4 University of Oxford, Oxford, United Kingdom; 5 Université de Neuchâtel, Neuchâtel, Switzerland; Public Library of Science, UNITED KINGDOM

## Abstract

We introduce “EloChoice”, a package for R which uses Elo rating to assess pairwise comparisons between stimuli in order to measure perceived stimulus characteristics. To demonstrate the package and compare results from forced choice pairwise comparisons to those from more standard single stimulus rating tasks using Likert (or Likert-type) items, we investigated perceptions of physical strength from images of male bodies. The stimulus set comprised images of 82 men standing on a raised platform with minimal clothing. Strength-related anthropometrics and grip strength measurements were available for each man in the set. UK laboratory participants (Study 1) and US online participants (Study 2) viewed all images in both a Likert rating task, to collect mean Likert scores, and a pairwise comparison task, to calculate Elo, mean Elo (mElo), and Bradley-Terry scores. Within both studies, Likert, Elo and Bradley-Terry scores were closely correlated to mElo scores (all *r*s > 0.95), and all measures were correlated with stimulus grip strength (all *r*s > 0.38) and body size (all *r*s > 0.59). However, mElo scores were less variable than Elo scores and were hundreds of times quicker to compute than Bradley-Terry scores. Responses in pairwise comparison trials were 2/3 quicker than in Likert tasks, indicating that participants found pairwise comparisons to be easier. In addition, mElo scores generated from a data set with half the participants randomly excluded produced very comparable results to those produced with Likert scores from the full participant set, indicating that researchers require fewer participants when using pairwise comparisons.

## Introduction

### Likert-type rating vs pairwise comparisons

When a researcher wants to quantify some perceived characteristic for stimuli within a set, one way to achieve this is to ask participants to rate each stimulus along an integer scale, anchored on either end with bivalent labels. For example, a rater could be asked to evaluate the attractiveness (“characteristic”) of an image depicting a face (i.e. “stimulus”) along a gradient between not attractive at all and highly attractive that could be scored along integers between 1 and 7. This is often referred to as a Likert scale [1–3] but this is technically inaccurate as Likert himself used “scale” to refer to a group of multiple such items [4]. Discrete Visual Analogue Scale is suggested as a general alternative [5], but we will simply refer to the practice as “Likert rating”. When inter-rater agreement is high, and average responses are calculated from a reasonable sample of raters, Likert rating tends to produce repeatable results that convey useful information about the stimuli [6]. This represents a mathematically straight-forward process that is easy for researchers with even minimal technical ability to complete using very basic and readily available hardware and software tools.

From an individual participants’ perspective, however, the rating experience could be better. The scale itself can be a challenging concept for users with a weak grasp of number lines; for instance, small children [7], or members of some traditional, pre-literate cultures [8]. More generally, using a rating scale can increase cognitive demand as it requires conscientious raters to monitor their responses to each stimuli and retrospectively compare them to responses from previous perceptive states, whereas paired comparisons imposes no such constraint [9].

One alternative to Likert rating is to show stimuli in pairs and ask participants to choose which one better expresses some characteristic, for example, which of two faces is more attractive. The results of these pairwise comparisons can be used to quantify perceptions of a stimulus according to a mathematical model that calculates its probability of being judged to be more or less expressive than a given set of alternatives. This method was first developed in the context of psychological research by Thurstone [10], but developed further by Bradley & Terry [11]. A class of mathematically-related models are now often known as Bradley-Terry models [12–13], and include a variety of sophisticated extensions and approaches to model-fitting [9, 14–16]. Also related are models that were independently derived to rank chess players—the earliest by Zermelo [17] and the most famous by Elo [18], which in turn has led to more complex derivatives such as Glicko [19] and TrueSkill^™^ [20].

There are a number of advantages associated with pairwise comparisons. They are less complicated for participants: there are only two choices for each decision, the choices do not require an understanding of number lines, and there is no need to track responses from previous decisions. Besides this, each decision made provides information about two stimuli, therefore representing a more efficient use of participant time. In previous contrasts, results from Likert ratings correlate strongly with pairwise comparison methods [21–22]. Nevertheless, pairwise comparisons are much less used, perhaps because many Bradley-Terry models, although simple by the standards of mathematicians, are comparatively complex and computationally intensive. However, this is no longer a serious constraint as the hardware and software capabilities to overcome it are readily available. Elo rating, in particular, is a relatively simple approach that offers conceptual accessibility, widespread familiarity and use [23–27], and recent programming implementations [28–29]. The purpose of this paper is to demonstrate the application of Elo rating to pairwise comparisons of stimuli, and introduce “EloChoice”, an R package optimized for this use and designed to be accessible even to those with limited experience of the free, and increasingly popular, R programming environment [30]. The package including its source code is available from https://cran.r-project.org/web/packages/EloChoice/index.html and includes a detailed manual (https://cran.r-project.org/web/packages/EloChoice/vignettes/tutorial.pdf).

### Elo rating

The following paragraphs outline the Elo rating process in brief, but further details and more in-depth discussion are readily available [18, 23, 31, 29].

Elo rating was developed to quantify and track over time skill distribution amongst chess players using information available from their previous matches. After a match between two players, points are exchanged depending on both players’ prior probability of winning the match. Each player enters with a score based on their match records, and the disparity between scores is used to predict the probability of outcomes, according to the following formulas, which assume a logistical distribution [18]:

$$E_{1}=\frac{1}{1+10^{\left(S_{2}-S_{1}\right)/400}}$$ (1)

$$E_{2}=\frac{1}{1+10^{\left(S_{1}-S_{2}\right)/400}}$$ (2)

where *E*_1_ and *E*_2_ are the estimated probabilities of a win for players 1 and 2 respectively, and *S*_1_ and *S*_2_ are their current scores. Players with equal scores are expected to be equally likely to win. After each new match, more information is available and is used to update each player’s scores, according to the following formulas:

$${S\prime }_{1}=S_{1}+k(O_{1}-E_{1})$$ (3)

$${S\prime }_{2}=S_{2}+k(O_{2}-E_{2})$$ (4)

where *O*_1_ and *O*_2_ are the actual outcomes for players 1 and 2 respectively (win = 1, loss = 0), *S*’_1_ and *S*’_2_ are their new scores, and *k* is a constant representing maximum point exchange. The exact value of *k* affects how quickly scores change and their eventual range, but, given a sufficient number of matches, will have a limited effect on player rankings [29, 31–32].

A key feature of the system is that the less expected the win, the more scores change (both for winner and loser). An unsurprising victory over a far weaker opponent will not result in a large score change, but an upset win will. Therefore, the highest scores require wins over most other opponents, including those with above-average ability.

Elo rating can be readily adapted to quantify the distribution of perceived attributes for a set of stimuli on an interval scale, allowing simple side-by-side comparisons while taking into account disparity between stimuli. Each pairwise comparison can be seen as a contest between two stimuli, with the one chosen as a better exemplar of some attribute being the winner. All stimuli begin with the same starting score, but diverge as successive comparisons take place. Because the number of points exchanged in any comparison is symmetrical (the winner’s gains equal the loser’s losses), the mean score will always equal the starting score, so long as the number of stimuli in the set remains stable. When stimuli are paired randomly, the scores should quickly converge to a stable ranking, assuming sufficient variation among stimuli and a reasonable degree of shared perceptions among rating participants. As with chess players, there is no requirement that all combinations of stimuli are observed, and, indeed, this is one of the system’s chief advantages [33].

There are at least two problems associated with the Elo rating system in the context of this application, but both are readily surmountable. The first problem arises from the fact that final scores can be influenced by the sequence order of contests, because the same contest outcomes will produce different results depending on the order in which they are observed. This is appropriate when the targets being scored are entities that may become weaker or stronger with time (such as players gaining or losing skill), but psychological stimuli are typically static in this regard and hence the order of events is not capturing any useful information. As there is nothing special about the sequence of contests presented to participants, any variation in final scores introduced by sequence variation is undesirable noise. To counter this problem, we propose a simple fix; randomly shuffle the original sequence presented to participants multiple times to create multiple virtual sequences, and average Elo scores for each stimuli from the different sequences to generate mean Elo scores that are free from sequence noise.

A second problem is that there is currently no commonly accepted way to measure consistency across paired comparison trials. It is often desirable to measure the consistency of ratings for stimuli across multiple raters (to gauge the extent to which raters share perceptions about targets), and in the case of Likert rating this is most appropriately achieved by calculating the intraclass correlation coefficient (ICC) [34–35]. To provide a conceptually (but not directly) comparable value, our solution is to propose a novel measure, the Elo consistency index, which tracks how often outcomes deviate from predictions based on previous judgments (that is, those trials in which the stimulus with the lower Elo score was chosen–in other words, an upset or reversal). Because the preceding Elo score is generated by the judgements made in previous comparisons, violations of expected outcomes can be seen as inconsistency between judgements. This inconsistency can occur both between and within raters. The index will be biased towards tracking one form of inconsistency or the other depending on the ratio of raters to the times each rater makes a judgement on each stimulus. In the case that each rater makes only one judgement on each stimulus, the index will be entirely tracking between-rater consistency (because each preceding Elo score was generated by judgements made by previous raters only). The index can be calculated for any sequence of trials (across any number of raters) and is defined by the following formula:

$$R=1-\frac{\sum_{i=1}^{N}u_{i}}{N}$$ (5)

where *u* is a vector of 1’s and 0’s, in which a 1 indicates that the expected outcome was violated and a 0 indicates that it was not, and *N* is the total number of trials for which an expectation existed (that is, for all trials in which the preceding Elo score difference was not 0). The resulting index value will vary between 0 and 1. Index values approach 1 as fewer expectation violations (or upsets/reversals) are observed, and approach 0 as more are observed. An index value of 0.5 indicates random choice, but values of less than 0.5 are technically possible. Index values for each sequence generated can be averaged to produce a mean consistency index score to correspond with mean Elo scores.

A weighted version of the Elo consistency index can be calculated by taking into account the score disparity observed in expectation violations, according to the following formula:

$$R\prime =1-\sum_{i=1}^{N}\frac{u_{i}*w_{i}}{\sum w}$$ (6)

where *u* is the same vector described above and *w* is the absolute difference in preceding Elo scores between members of a trial. This can be understood as the proportion of all points exchanged in a sequence that were correctly predicted by preceding Elo scores. The weighted consistency index will again vary between 0 and 1, and larger expectation violations (that is, where the difference in preceding Elo scores is greater) will negatively impact values of *R* more. Generally the weighted consistency index value is expected to be greater than the unweighted value, as the magnitude of difference between Elo scores should negatively predict probability of an expectation violation.

To demonstrate Elo rating in action and to compare it both to Likert rating and to a basic Bradley-Terry model, we use the example of perceived physical strength from stimuli composed of images of human male bodies. Previous research using Likert-type ratings have shown that participants judging strength from photographs of men’s bodies display high inter-rater agreement, and mean ratings correlate robustly with men’s actual measured strength and strength-related body measurements [36]. We use both laboratory (Study 1) and online participants (Study 2) to demonstrate that the Elo-rating method is appropriate in both contexts.

## Study 1—Laboratory rating

### Methods

#### Target stimuli

Stimuli consisted of digital images (399 x 710 pixels) of 82 men (*mean* age = 21.4, *sd* = 2.5, min = 18, max = 30) depicted standing on a raised platform and facing the camera. The men are wearing only a pair of black boxer briefs. Their heads are digitally blurred, making positive identification from face alone impossible. Images were all captured from the same camera, mounted on a tripod at the same height, location and orientation. Anthropometric measures were collected from each man on the day he was image was captured. The men who posed for the images gave written informed consent beforehand, and were reimbursed for their time (£40 GBP). Approval for their participation was given by the Faculty of Science Human Research Ethics Committee, at the University of Bristol.

#### Stimuli measures

We used hand grip strength as an assay of overall physical strength, as it easy to collect and previous research has shown it to be highly correlated with other measures, such as chest and shoulder strength [36]. Each man who posed for stimulus images was instructed to squeeze a hand dynamometer as hard as he could using one hand. Trials were completed for both the right hand and the left hand separately. For each man, the average of these two squeezes (measured in kg) was taken and used as the grip strength score associated with the stimulus image (*mean* = 39.5, *sd* = 7.1).

Body size has also been found to correlate with upper-body strength in males [36]. We used six anthropometric measures to construct a proxy of body size: height, weight, shoulder circumference, chest circumference, bicep circumference and forearm circumference. We performed a principal components analysis on these measures and used scores for the first principal component (explaining 72.4% of total variance) as our body size score (PC1 of body size; *mean* = 0, *sd* = 2.1). Grip strength and PC1 of body size were moderately correlated (*r* = 0.46).

#### Rating participants

56 participants (28 male, 28 female; *mean* age = 19.9, *sd* = 1.98) took part in the rating study. These participants were students recruited from the University of Bristol either by opportunity sampling (word of mouth) or for partial course credit in the undergraduate Psychology programme. Participants gave written informed consent before completing any task. Approval for their participation was given by the Faculty of Science Human Research Ethics Committee, at the University of Bristol.

#### Rating procedure

Participants viewed each image three times, once within a Likert rating task, and twice within a paired comparison task. The order of these tasks was counterbalanced. The tasks were presented on a laboratory computer using e-Prime software. Each participant completed the tasks in private, and no time requirement was specified.

For the Likert rating task, images were presented sequentially in random order. Each image was presented for 2 seconds before text appeared, reading, “How strong is this man?” accompanied by a 7-point scale anchored by “very weak” at point 1 and “very strong” at point 7. Participants used the keyboard to indicate a response, and time to make a response was recorded. 82 responses were required to complete the task.

The paired comparison task was presented in two blocks. In each block, each image was randomly paired with a different image and each pair was presented in random order. The paired images were presented side by side. Each pair was presented for 2 seconds before text appeared, reading, “Which man is stronger?”. Participants used the keyboard to indicate a response by pressing ‘z’ to choose the image on the left, and ‘m’ to choose the image on the right. Again, response time was recorded. 41 responses were required to complete each block, and 82 responses were required to complete the task.

#### Rating measures

For each stimulus image, Likert responses from all rating participants were averaged to generate a mean Likert rating score.

To generate Elo scores for each stimulus image, all paired comparison trials were arranged in their original sequence, ordered first by rating participant number and then by trial number. Beginning Elo scores were set to 0, and *k* was arbitrarily set to 100, following precedent [29]. Using Formulas 1–4, updates to Elo scores were calculated for each trial sequentially—each trial involved updating scores for two images. Final Elo scores were the scores for each image after the calculation performed for the last trial within the sequence.

Elo scores were calculated for each image using the original sequence, as described above, and then for 99 randomized trial sequences. Final Elo scores from each of the 100 sequences were averaged together to generate mean Elo (mElo) scores for each image. For comparative purposes, an additional set of mElo scores was generated by averaging Elo scores across 1000 sequences (the original sequence plus 999 randomized sequences). These are referred to as mElo(M) scores in the subsequent analyses.

Scores were collected by extracting worth parameters from a Bradley-Terry model fit using the paired comparison data. As these scores are highly skewed, they are log-transformed for the purposes of correlations.

#### Statistical software

All analyses were conducted in R version 3.2.1 [30].

Principal components analysis was conducted using the “FactoMineR” package [37].

Elo and mElo scores and the Elo consistency indices were calculated using the “EloChoice” package [38].

Bradley-Terry models were fit and worth parameters extracted using the “psychotools” package [39].

Comparisons between overlapping and non-overlapping correlations from dependent groups were conducted using the “cocor” package [40] and Williams’s *t* [41].

Intraclass correlation coefficients were calculated using “irr” package [42].

Comparisons between means from dependent groups were conducted using the “perfect-t-test” script [43].

### Results

All analyses shown incorporate responses from male and female rating participants together, as we were not interested in sex differences for this project.

#### Descriptive statistics of rating measures

Table 1 displays descriptive statistics for Likert ratings of perceived strength, and Elo scores (from the original sequence), mElo scores, mElo(M) scores, and Bradley-Terry scores from perceived strength comparisons for the sample of 82 stimulus images.

**Table 1:** Descriptive statistics for mean Likert ratings, Elo scores, mElo scores (using 100 iterations), mElo(M) scores (using 1000 iterations) and Bradley-Terry scores for the 82 stimulus images.

	*mean*	*standard deviation*	*median*	*minimum*	*maximum*
Likert	3.97	0.90	3.89	2.14	5.91
Elo	0	289.5	-11	-727	755
mElo	0	292.8	-29.6	-686.2	628.3
mElo(M)	0	292.2	-33.2	-672.7	635.0
Bradley-Terry	0.012	0.024	0.0028	0.000085	0.15

#### Correlations between rating measures and stimuli measures

All rating measures were strongly correlated with each other (see Table 2), particularly in the cases of mElo, mElo(M) and Bradley-Terry scores (log-transformed), which were almost perfect correlated (all *r*s > 0.999).

**Table 2:** Correlations between mean Likert ratings, Elo scores, mElo scores (100 iterations), mElo(M) scores (1000 iterations), and log-transformed Bradley-Terry scores for the 82 stimulus images.

	Likert	Elo	mElo	mElo(M)
Likert	-			
Elo	0.91	-		
mElo	0.96	0.95	-	
mElo(M)	0.96	0.95	1	-
Bradley-Terry	0.96	0.95	1	1

All ratings measures were correlated with stimuli measures (grip strength and PC1 of body size) to a similar extent (see Table 3), although these relationships were weakest for mean Likert ratings.

**Table 3:** Correlations between stimuli measures (grip strength and PC1 of body size) and rating measures (mean Likert ratings, Elo scores, mElo scores (100 iterations), mElo(M) scores (1000 iterations), and log-transformed Bradley-Terry scores) for the 82 stimulus images.

	Likert	Elo	mElo	mElo(M)	Bradley-Terry
grip strength	0.4	0.43	0.44	0.44	0.45
PC1 of body size	0.6	0.68	0.65	0.65	0.65

The very similar results obtained by mElo and mElo(M) suggest that there is little to be gained by generating mElo scores from greater than 100 sequences.

#### Comparing Elo and mElo scores

The 100 sequences used to calculate the mElo scores each produced their own set of Elo scores. Considered separately, each set significantly correlated with grip strength, but the strength of these correlations varied (0.35 < all *r*s < 0.49). This was also the case for correlations with PC1 of body size (0.56 < all *r*s < 0.68). These 100 sets of Elo scores were all strongly correlated with each other, but again with considerable variation (0.86 < all *r*s < 0.95).

**Fig 1:**
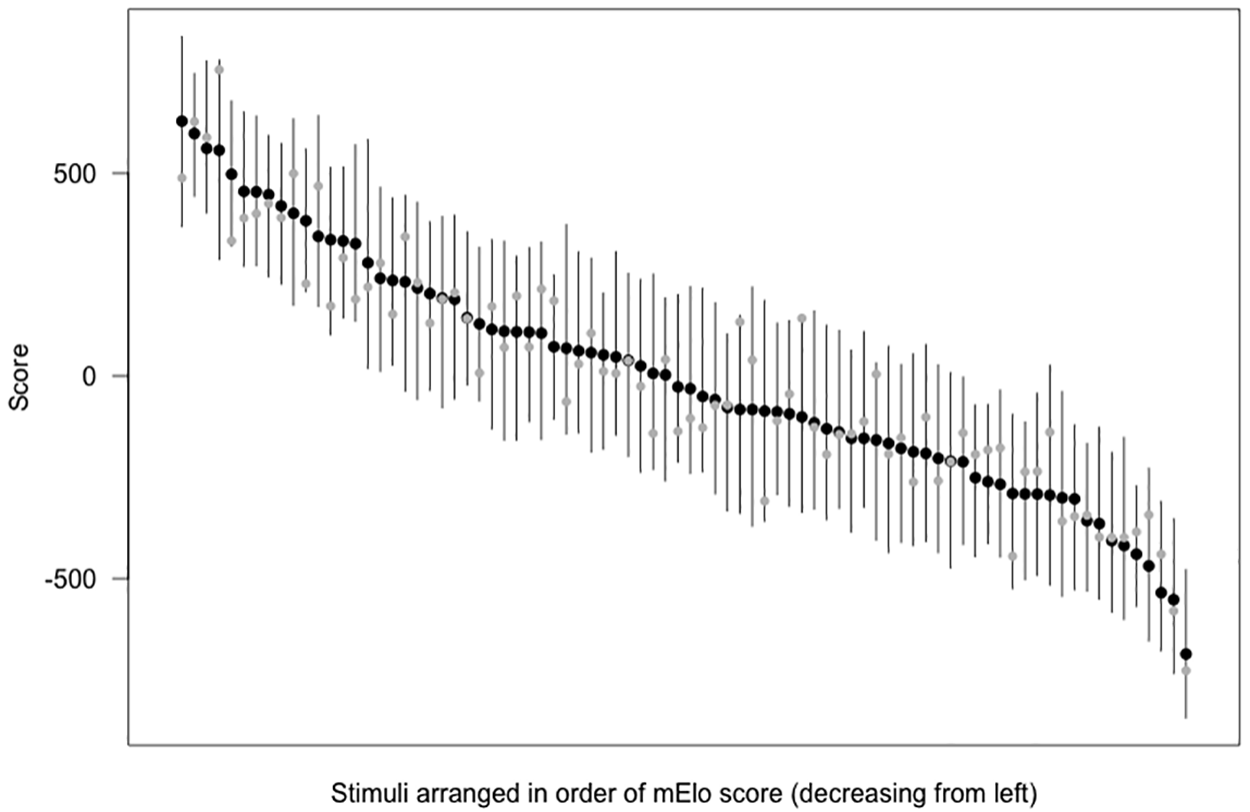
Elo scores from the original sequence (grey circles) and mElo scores (black circles) are depicted for each of the stimulus images, arranged from left to right in order of decreasing mElo score. The whiskers depict the range of Elo scores from the 100 sequences generated to calculate the mElo scores.

Fig 1 shows Elo scores from the original sequence and mElo scores for individual stimulus images on the same plot for comparison.

#### Comparing Likert ratings and mElo scores from reduced data sets

To show how the relationships between measures of perceived strength and stimulus variables is affected by the number of raters, Likert ratings of perceived strength and mElo scores from perceived strength comparisons were recalculated using just the first half of rating participants (*n* = 28; 14 males and 14 females), and then again using just the first quarter of rating participants (*n* = 14; 7 males and 7 females). Correlation coefficients (*r*s) for these relationships using the full, half, and quarter participant sets are show in Table 4.

**Table 4:** Correlation coefficients for relationships between Likert ratings and mElo scores and stimulus variables for full, half and quarter participant sets.

	Likert ratings		mElo scores	
	grip strength	PC1 of body size	grip strength	PC1 of body size
full set	0.40	0.60	0.44	0.65
½ set	0.37	0.57	0.41	0.60
¼ set	0.32	0.53	0.38	0.57

For the Likert ratings, correlations from the halved set were significantly smaller than those from the full set (grip strength: *t* = -2.100, *df* = 79, *p* = 0.039*; PC1 of body size: *t* = -2.412, *df* = 79, *p* = 0.018*), and correlations from the quartered set were significantly smaller than those from the halved set (grip strength: *t* = -3.616, *df* = 79, *p* < 0.001***; PC1 of body size: *t* = -3.203, *df* = 79, *p* = 0.004**).

For the mElo scores, correlations from the halved set were significantly smaller than those from the full set (grip strength: *t* = -2.144, *df* = 79, *p* = 0.035*; PC1 of body size: *t* = -4.524, *df* = 79, *p* < 0.001***), but correlations from the quartered set were not significantly smaller than those from the halved set.

#### Consistency of Likert ratings and mElo scores

To measure inter-rater consistency of Likert ratings, an average score intraclass correlation coefficient (model: two-way; type: consistency) was calculated [34]. The result (*ICC* = 0.984, 95% CI: 0.979 < *ICC* < 0.989) indicates very high agreement between raters about the perceived strength of targets.

To measure inter-trial consistency of perceived strength comparisons used to calculate mElo scores, we used the novel consistency indices described (formulas 5 and 6). The mean unweighted consistency index was 0.766, while the mean weighted consistency index was 0.864. It is important to note that these values are not directly comparable with ICC values. The unweighted value indicates that 76.6% of trial outcomes were concordant with the predictions of preceding Elo scores, and the weighted value indicates that the direction of 86.4% of all points exchanged was predicted by preceding Elo scores. Both values indicate high consistency of judgements between trials.

#### How many raters are needed to establish stable consistency indices?

**Fig 2:**
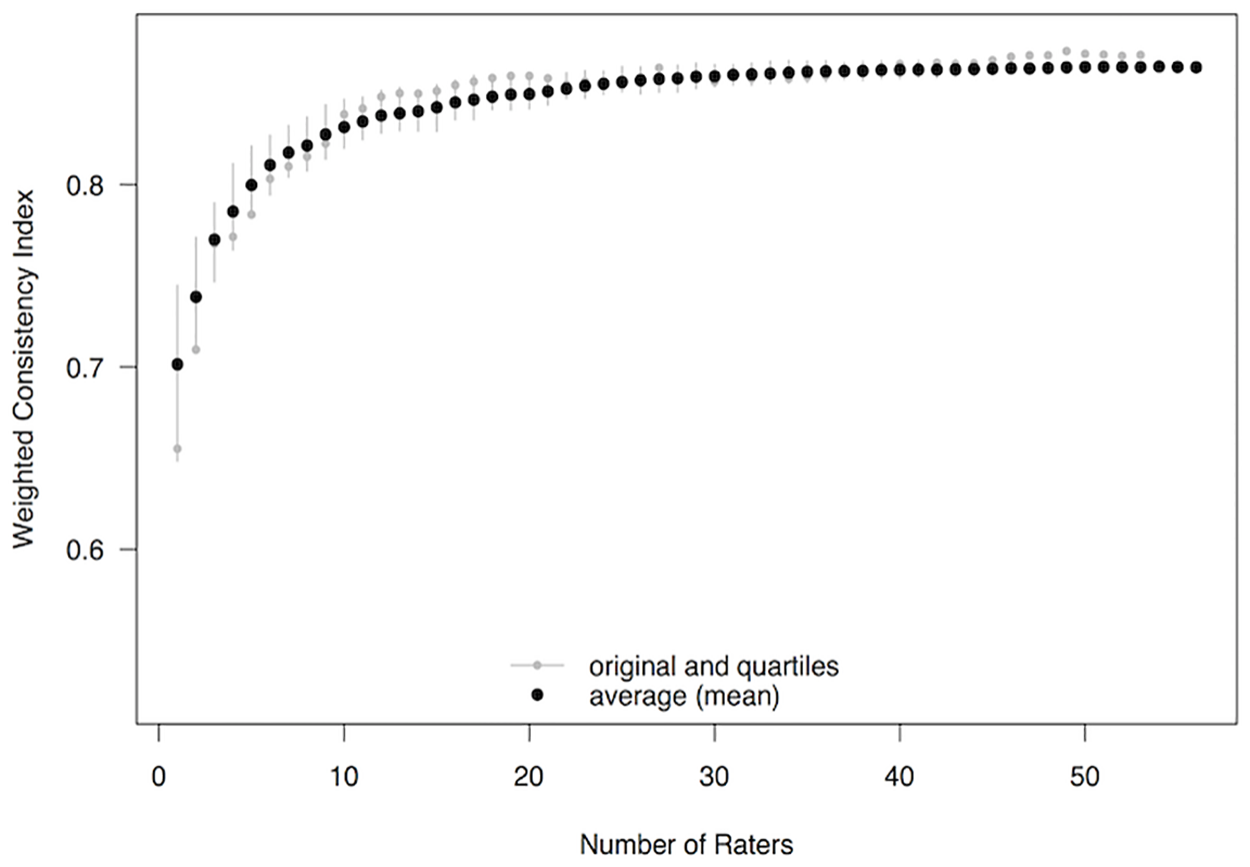
Weighted consistency indices for the original sequence (small grey circles) and mean weighted consistency indices (large black circles) calculated for an increasing number of Study 1 raters (1–56, in increments of 1). The whiskers represent inter-quartile ranges of indices from the 100 sequences generated to calculate the mean index.

Values for the consistency indices are expected to become more accurate as the number of raters increases. This is because the number of trials with an expected result will increase, effectively increasing the sample size from which to estimate consistency. To demonstrate how many raters are needed before these estimates become stable, the weighted consistency index was re-calculated multiple times starting with data from just the first rater (according to the original sequence), then adding the second rater, then the third rater, and so on until all 56 raters were included. Fig 2 depicts these results, showing that index values reached a stable plateau after roughly 30 raters. This could also be used as an indication of the minimal number of raters required to produce relatively stable rankings of mElo scores.

#### Comparing decision times for Likert ratings and paired comparison tasks

Mean decision times (in milliseconds) for Likert ratings (*mean* = 995.6, *sd* = 392.6) were compared to mean decision times (in milliseconds) for paired comparisons (*mean* = 635.8, *sd* = 233.7) using a paired t-test. The result indicates a significant difference between the two (*t*_*55*_ = 7.65, *p* < 0.001***), and a large effect size (Hedges’ *g* = 1.1, 95% CI: 0.76 < *g* < 1.46).

**Fig 3:**
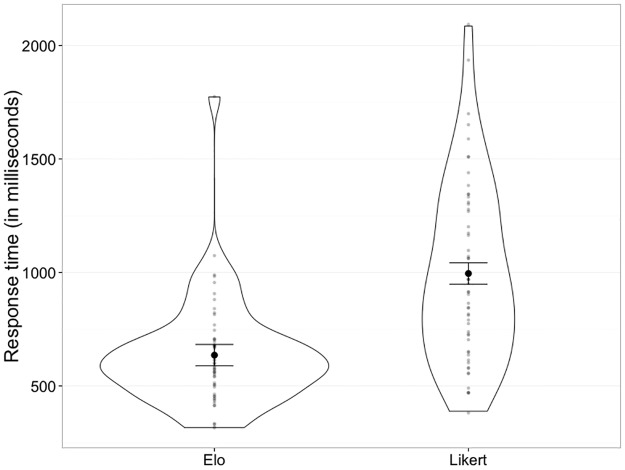
Violin plots depict the distribution of response times for paired comparison trials (left), and Likert trials (right). Individual values are depicted by small grey circles. Error bars depict within-subject 95% confidence intervals. Means are depicted by large black circles.

Fig 3 shows mean decision times for Likert ratings and paired comparisons.

#### Comparing computation times for mElo and Bradley-Terry scores

Both mElo and Bradley-Terry models involve more computation than either Likert or Elo. To compare the time it takes to compute scores via these methods, we timed each procedure on the same laptop computer (2.2 GHz processor speed, 8 GB RAM), using R’s system.time function. The elapsed time for computing mElo scores for the entire data set was 3 seconds. The elapsed time for computing Bradley-Terry scores for the entire data set was 1017 seconds.

## Study 2—Online rating

The results of Study 1 suggest that mElo scores correspond well with Likert ratings for tasks completed within the lab, but with the advantages offered by web-based data collection (speed, cost, diversity of participants) it is important to check whether comparable results are obtained for tasks completed online. Previous research indicates that online performance is generally good compared to performance in labs [44] and that attention to instructions is actually better [45].

### Methods

Target stimuli, stimuli measures, rating measures and statistical software were identical to Study 1, but mElo(M) scores (based on 1000 sequences) were not calculated.

#### Rating participants

96 participants (59 male, 37 female; *mean* age = 32.6, *sd* = 9.31) took part in and completed the rating study. These participants were recruited from mTurk (US residents only) and paid $**3** USD for participation. Before completing any task, participants gave informed consent by ticking a check box stating that they had read and understood the procedure. Approval for their participation was given by the Faculty of Science Human Research Ethics Committee, at the University of Bristol.

#### Rating procedure

Participants viewed each image three times, once within a Likert rating task, and twice within a paired comparison task. The order of these tasks was counterbalanced. The tasks were presented using Xperiment online presentation software (www.experiment.mobi). Unlike Study 1, no response times were recorded.

For the Likert rating task, images were presented sequentially in random order. Each image was presented for 2 seconds before text appeared, reading, “How strong is this man?” accompanied by a 7-point scale anchored by “very weak” at point 1 and “very strong” at point 7. Participants clicked a point on the scale to indicate a response. 82 responses were required to complete the task.

The paired comparison task was presented in two blocks. In each block, each image was randomly paired with a different image and each pair was presented in random order. The paired images were presented side by side for 2 seconds before text appeared, reading, “Which man is stronger?”. Participants indicated a response by clicking on the button corresponding to either the left or right image. 41 responses were required to complete each block, and 82 responses were required to complete the task.

### Results

All analyses shown incorporate responses from male and female rating participants together, as we were not interested in sex differences for this project.

#### Descriptive statistics

Table 5 displays descriptive statistics for Likert ratings of perceived strength, and Elo scores (from the original sequence), mElo scores, and Bradley-Terry scores for the sample of 82 stimulus images.

**Table 5:** Descriptive statistics for mean Likert ratings, Elo scores, mElo scores and Bradley-Terry scores for the 82 stimulus images.

	*mean*	*standard deviation*	*median*	*minimum*	*maximum*
Likert	3.90	0.79	3.76	2.16	5.65
Elo	0	277.8	-34	-571	628
mElo	0	255.8	-20.7	-620.2	562.4
Bradley-Terry	0.012	0.019	0.0044	0.00019	0.096

#### Correlations between rating measures and stimuli measures

All rating measures were strongly correlated with each other (see Table 6), particularly mElo and Bradley-Terry scores (log-transformed), which were almost perfect correlated (*r* = 0.9992).

**Table 6:** Correlations between mean Likert ratings, Elo scores, mElo scores and log-transformed Bradley-Terry scores for the 82 stimulus images.

	Likert	Elo	mElo
Likert	-		
Elo	0.92	-	
mElo	0.97	0.95	-
Bradley-Terry	0.97	0.94	1

All ratings measures were correlated with stimuli measures (grip strength and PC1 of body size) to a similar extent (see Table 7).

**Table 7:** Correlations between stimuli measures (grip strength and PC1 of body size) and rating measures (mean Likert ratings, Elo scores, mElo scores and log-transformed Bradley-Terry scores) for the 82 stimulus images.

	Likert	Elo	mElo	Bradley-Terry
grip strength	0.38	0.40	0.43	0.43
PC1 of body size	0.61	0.59	0.62	0.62

#### Correlations between rating measures from Study 1 and Study 2

To examine whether lab participants and online participants produce similar results using these rating measures, we correlated ratings and scores from Studies 1 and 2 (see Table 8). The measures were strongly correlated with one another, indicating that mode of delivery (lab or online) did not have a great impact.

**Table 8:** Correlations between rating measure scores from Study 1 and Study 2.

		Study 2			
		Likert	Elo	mElo	Bradley-Terry
Study 1	Likert	**0.98**			
	Elo	0.91	**0.85**		
	mElo	0.96	0.91	**0.97**	
	Bradley-Terry	0.96	0.92	0.97	**0.97**

#### Comparing Elo and mElo scores

**Fig 4:**
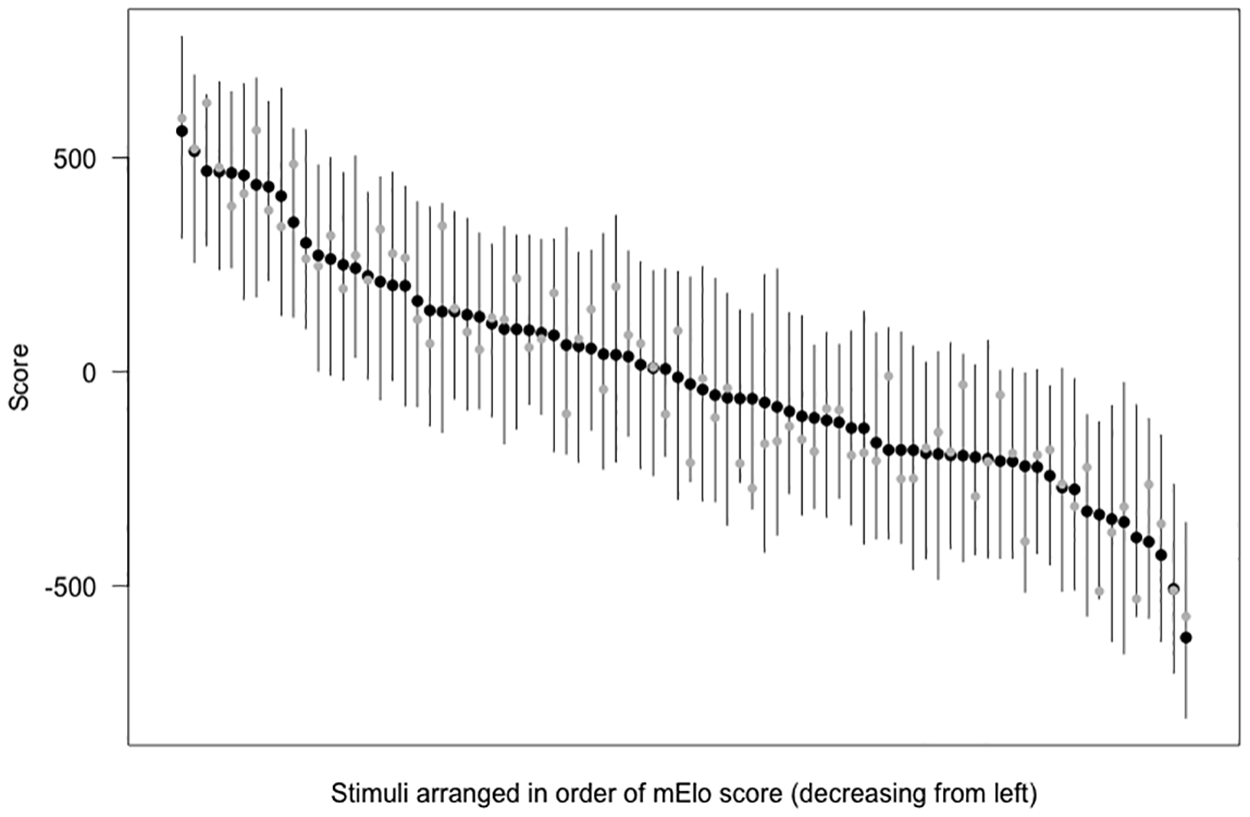
Elo scores from the original sequence (grey circles) and mElo scores (black circles) are depicted for each of the stimulus images, arranged from left to right in order of decreasing mElo. The whiskers depict the range of Elo scores from the 100 sequences generated to calculate the mElo scores.

Fig 4 shows Elo scores from the original sequence and mElo scores for individual stimulus images on the same plot for comparison.

#### Consistency of Likert ratings and mElo scores

To measure inter-rater consistency of Likert ratings, an average score intraclass correlation coefficient (model: two-way; type: consistency) was calculated. The result (*ICC* = 0.99, 95% CI: 0.985 < *ICC* < 0.994) indicates very high agreement between raters about the perceived strength of targets.

To measure inter-trial consistency of perceived strength comparisons used to calculate mElo scores, we used the novel consistency indices described (formulas 5 and 6). The mean unweighted consistency index was 0.740, while the mean weighted consistency index was 0.837. Again, note that these values are not directly comparable with ICC values. The unweighted value indicates that 74.0% of trial outcomes were concordant with the predictions of preceding Elo scores, and the weighted value indicates that the direction of 83.7% of all points exchanged was predicted by preceding Elo scores. Both values indicate high consistency of judgements between trials.

#### How many raters are needed to establish stable consistency indices?

**Fig 5:**
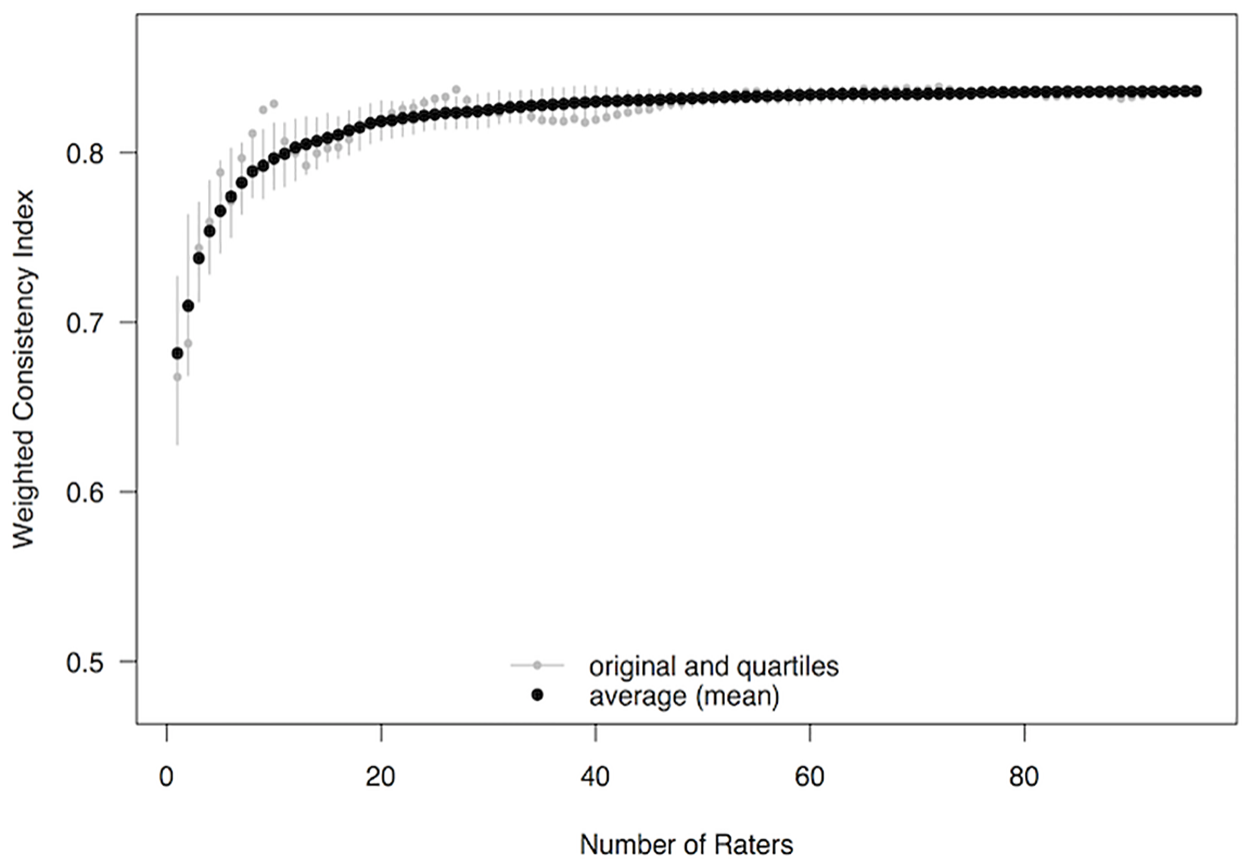
Weighted consistency indices for the original sequence (small grey circles) and mean weighted consistency indices (large black circles) calculated for an increasing number of Study 1 raters (1–96, in increments of 1). The whiskers represent inter-quartile ranges of indices from the 100 sequences generated to calculate the mean index.

As in Study 1, to demonstrate how many raters are needed before estimates of consistency become stable, the weighted consistency index was re-calculated multiple times, starting with data from just the first rater (according to the original sequence), then adding the second rater, then the third rater, and so on until all 96 raters were included. Fig 5 depicts these results, showing that index values changed little after about 40 raters. Again, this could also be used as an indication of the minimal number of raters required to produce relatively stable rankings of mElo scores.

#### Comparing computation times for Bradley-Terry and mElo scores

To compare the time it takes to compute scores via mElo and Bradley-Terry methods, we timed each procedure on the same laptop computer (2.2 GHz processor speed, 8 GB RAM), using R’s system.time function. The elapsed time for computing mElo scores for the entire data set was 5 seconds. The elapsed time for computing Bradley-Terry scores for the entire data set was 904 seconds.

## Discussion

Likert ratings, Elo scores and Bradley-Terry scores were closely correlated with mElo scores, indicating that they were tracking perceptions similarly. Each measure of perceived strength was also similarly correlated with actual strength and strength-related measurements of the men depicted in the stimuli, indicating that these perceptions reliably track actual physical differences between men, as reported by Sell et al. [36]. In addition, these relationships were observed in both the laboratory, using a primarily UK undergraduate participant base, and online, using a more diversely aged US participant base. On the basis of these facts alone, there is little to separate one measure from another, and little reason to favour one above the other options. However, we argue that mElo scores represent a good alternative option to Elo scores, Bradley-Terry scores and Likert ratings, for distinct reasons.

Figs 1 and 4 demonstrates the range of Elo scores that can result from different sequences of the same trial comparisons with the same outcomes, and the range of correlations with grip strength and PC1 of body size (Study 1) demonstrates why this is problematic. As argued earlier, this variation in final scores represents an undesirable feature in the context of our rating temporally stable features, and we proposed mElo scores to address this issue. When multiple mElo scores are generated, each using the same trial data but using different randomly shuffled sequence orders, the results are far less variable. Each mElo score was almost perfectly correlated with all others, and there was no detectable variation between them when predicting actual strength and strength-related measurements. This was even the case for the one mElo score that was generated using 1000 different sequence orders, which indicates that, in this case at least, using 100 sequence orders is sufficient and saves computing time without cost.

We argue that the mElo approach is conceptually simpler than standard Bradley-Terry models, and thus more likely to appeal to researchers who are new to pairwise comparison. Our results suggest that mElo is computationally simpler as well, calculating score in 5 seconds or less, whereas Bradley-Terry models took over 15 minutes. This could be related to the number of stimuli used in our example, so studies using an even greater number of stimuli may suffer even greater time disparities.

Although results from Likert ratings and mElo scores were largely similar for this task, the procedure for obtaining them was quite different, leading to a very divergent participant experience. Participants completed pairwise comparison trials significantly quicker than rating trials, and the effect size for this difference was large (see Fig 3). Taking response time as a proxy of cognitive load [46], the speed difference between the trial types indicates that participants found the pairwise comparisons to be easier. This is likely to be a particularly important consideration for more difficult tasks (e.g. rating characteristics which are not readily apparent, such as personality traits from faces [47], or kinship in monkeys [48]), or for participant groups that have either compromised capacity or limited experience with stimulus tasks, particularly those involving novel concepts.

In addition, because participants complete pairwise comparison tasks more quickly, they might be willing to be paid less to provide similarly useful information. This is not the only way in which pairwise comparisons may prove to be more efficient. Note that correlations with actual strength and strength-related measures deteriorated as more raters were excluded for both Likert ratings and mElo scores, despite strong correlations with ratings and scores from the full participant set. This indicates that collecting data from more participants is likely to beneficial for both methods. However, also note that the mElo scores obtained from the halved set had strikingly similar associations with stimulus variables as Likert ratings from the full set did, and similarly mElo scores from the quartered set produced similar associations as Likert ratings from the halved set (see Table 2). If this pattern is typical of other data sets, this suggests that only half as many pairwise comparison participants are required to produce results equivalent to those from ratings tasks. This could represent a considerable savings for researchers both in time and participant payments.

In conclusion, using pairwise comparisons in conjunction with the “EloChoice” package represents a simple alternative option for researchers interested in quantifying perceived characteristics of stimuli, offering participants a less demanding experience while requiring fewer of them, and representing a minimal learning cost even for researchers inexperienced with R.
